# National Medical College

**Published:** 1849-03

**Authors:** 


*National Medical College.*—The third annual meeting of the American Medical Association is now near at hand. We have noticed the appointment of delegates by medical societies in different parts of the country, and it is to be presumed the attendance will be not less, if not larger, than heretofore. We speak not altogether without knowledge when we say, that our Boston brethren will be prepared to extend to delegates and members of the Association a cordial reception, and it will be the fault of the latter if the occasion be not, socially, at least, one of interest and gratification. As the success and prosperity of the Association depend upon the fullness with which the profession of the country is represented, it is to be hoped that delegates and members will endeavor to be present. Let representatives be selected who, in the first place, are eminently qualified by their capacity and zeal, and, in the second place, who will not fail to attend, unless prevented by extraordinary circumstances. A medical Congress will then be assembled from whose proceedings much good may be expected.

In view of the meeting in May next, it is well for reflecting members of the profession to consider what subjects may come before it, and what useful objects may be submitted for its action. It were desirable that plans to be proposed should be intimated before the meeting takes place, in order that they may, meanwhile, occupy the thoughts of the profession, and thus more matured opinions formed when the time for decision arrives. We do not make these remarks by way of preface to any project which we shall expect formally to introduce to the consideration of the Association, but we have been more and more forcibly impressed with the importance of an undertaking which we believe may be effected by the united efforts of the profession, in connection with the organization which now exists. We allude to a *National Medical College.* In one of the early numbers of the first volume of this Journal, we devoted some consideration to this subject. We would respectfully suggest it again, as a subject which, as it seems to us, is deserving, alike from its importance and feasibility, the attention of the profession.

By a *National Medical College,* we mean an institution of a higher order, and with more extensive scientific and educational objects, than can be embraced within the scope of the numerous existing schools of medicine in different sections of our country. Our idea is not to supersede the latter, but to fulfil ends which are now, and must of necessity continue to be, in a measure, beyond their reach. The purposes of such an institution as we should rejoice to see established, should be two fold: first, to provide, in the most ample and perfect manner, for the prosecution of each, and all the sciences relating, directly or collaterally, to medical knowledge—anatomy, human and comparative, special, histological, and microscopical; physiology in its most comprehensive signification; pathology; materia medica, botany and pharmacy; chemistry, general and animal; legal medicine, etc. etc. Second, to furnish complete courses of instruction in each, and all of these branches, with every available means and facilities for their elucidation. We would have, in other words, an institution which should, in the first place, combine all the circumstances most favorable to the successful cultivation of the various departments of medical science, and which would therefore conduce, in the highest degree, to the extension of medical knowledge: and, in the second place, an institution where all that is known, or might be developed in the progress of knowledge relating, directly or indirectly, to medical science, should be fully and adequately taught. It is sufficiently evident that no medical college now existing in this country (if in any other) affects to accomplish ends so wide and extensive; nor would it be desirable, if it were attainable, that all schools instituted to prepare students for entering on the practical duties of our profession, should attempt to compass them. The plan to be successfully carried out, must be a national one—it must be adopted by the general government, and sustained by unstinted appropriations from the national treasury.

We commend to the meditations of our readers the immense benefits which such an institution would be calculated to produce. Consider how it would conduce to the progress of scientific knowledge: how it would tend to elevate the character of the medical profession, by furnishing opportunities, as accessible as abundant, for the most profound and varied acquirements, and by raising the standard of qualifications for teachers in the medical colleges of the different states of the Union, and by its effect on our national medical literature! Consider, too, the position which American Medicine would be enabled to hold among other nations by such an exponent of its progressive and high character! We will not pursue the subject from these, and other points of contemplation, but leave it to be amplified by the reflections of our readers. If it strike their minds as it does us, it is a subject which is calculated to inspire the most animating and patriotic sentiments. The details of the plan we do not profess to have elaborated. This is to be done by the matured deliberations of combined intelligence and experience.

But it may be said, the enterprise is very well upon paper, but it is an air-castle—a chimera, which can never be realized. This incredulousness will perhaps be expressed by some who have no skepticism as to the propriety of undertaking the construction of a rail road to the Pacific! The truth is, the medical profession of the United States, having never exerted their united strength, members of it are apt to underestimate the power which the profession is capable of exerting. We believe, and, indeed, we have no doubt of the feasibility of the project. To effect it, the profession have only to resolve that it *may*, and *shall* be done. We assume that the General Government may constitutionally appropriate means to sustain a national institution of the character proposed. We suppose there need be no question as to this power. The question then is, can Congress be induced to do it? We answer that there is no room to doubt that they can be. In the project there is assuredly nothing sectional, nothing selfish. The objects are truly national, and in every respect consistent with the highest principles of civilization and philanthropy. Now, is it too much to think that the medical profession of this country can succeed in bringing our legislators to appreciate the character of the project, and to act accordingly? Let the American Medical Association, representing, as it does, the voice of the medical profession of the United States, agree upon a plan to be submitted, and let the members of the profession in the different states of the Union exert their influence, each in his own sphere, to bring about its adoption, and it will be done. We are aware that it is a common remark, and a just one, that legislation has heretofore done but little for the medical profession in this country, often, indeed, appearing to disregard its claims and interests. But why is this? It is because the profession has never exerted its combined influence to obtain measures of protection and fostering care. It must be obvious to every one, on a little reflection, that there is no body of men who are capable of effecting more through legislative action than medical men, if they were to unite for a common object, and choose to exert their combined influence to accomplish it. Union and effort alone are necessary. The profession of the whole country has now an organization in the American Medical Association. By means of this organization union may be secured, and through the instrumentality of its members in different states, the profession generally may be incited to exertion. Let us test the power which we can exercise by our collective, concentrated energies, in an effort to establish an Institution which will confer honor on the Association, the profession, and the country, and redound in an eminent degree to the interests of humanity!

